# Ischemic preconditioning reduces endoplasmic reticulum stress and upregulates hypoxia inducible factor-1α in ischemic kidney: the role of nitric oxide

**DOI:** 10.1186/1423-0127-19-7

**Published:** 2012-01-17

**Authors:** Asma Mahfoudh-Boussaid, Mohamed Amine Zaouali, Kaouther Hadj-Ayed, Abdel-Hédi Miled, Dalila Saidane-Mosbahi, Joan Rosello-Catafau, Hassen Ben Abdennebi

**Affiliations:** 1 Laboratory of human physiology, faculty of pharmacy, university of Monastir, Tunisia; 2 Hepatic ischemia reperfusion unit, Department of experimental pathology, Institut d'Investigacions Biomèdiques de Barcelona-Consejo Superior de Investigaciones Científicas, Barcelona, Spain; 3 Laboratory of biochemistry, faculty of pharmacy, university of Monastir, Tunisia

**Keywords:** kidney, ischemia-reperfusion, ischemic preconditioning, Akt, eNOS, HIF1-α, ER stress

## Abstract

**Background:**

Although recent studies indicate that renal ischemic preconditioning (IPC) protects the kidney from ischemia-reperfusion (I/R) injury, the precise protective mechanism remains unclear. In the current study, we investigated whether early IPC could upregulate hypoxia inducible transcription factor-1α (HIF-1α) expression and could reduce endoplasmic reticulum (ER) stress after renal I/R and whether pharmacological inhibition of nitric oxide (NO) production would abolish these protective effects.

**Methods:**

Kidneys of Wistar rats were subjected to 60 min of warm ischemia followed by 120 min of reperfusion (I/R group), or to 2 preceding cycles of 5 min ischemia and 5 min reperfusion (IPC group), or to intravenously injection of N^G^-nitro-L-arginine methylester (L-NAME, 5 mg/kg) 5 min before IPC (L-NAME+IPC group). The results of these experimental groups were compared to those of a sham-operated group. Sodium reabsorption rate, creatinine clearance, plasma lactate dehydrogenase (LDH) activity, tissues concentrations of malonedialdehyde (MDA), HIF-1α and nitrite/nitrate were determined. In addition, Western blot analyses were performed to identify the amounts of Akt, endothelial nitric oxide synthase (eNOS) and ER stress parameters.

**Results:**

IPC decreased cytolysis, lipid peroxidation and improved renal function. Parallely, IPC enhanced Akt phosphorylation, eNOS, nitrite/nitrate and HIF-1α levels as compared to I/R group. Moreover, our results showed that IPC increased the relative amounts of glucose-regulated protein 78 (GRP78) and decreased those of RNA activated protein kinase (PKR)-like ER kinase (PERK), activating transcription factor 4 (ATF4) and TNF-receptor-associated factor 2 (TRAF2) as judged to I/R group. However, pre treatment with L-NAME abolished these beneficial effects of IPC against renal I/R insults.

**Conclusion:**

These findings suggest that early IPC protects kidney against renal I/R injury via reducing oxidative and ER stresses. These effects are associated with phosphorylation of Akt, eNOS activation and NO production contributing thus to HIF-1α stabilization. The beneficial impact of IPC was abolished when NO production is inhibited before IPC application.

## Background

Ischemia-reperfusion (I/R) injury, commonly seen in the field of renal surgery or transplantation, is the leading cause of acute renal failure [1]. Ischemic preconditioning (IPC), defined as brief intermittent episodes of ischemia alternating with reperfusion, increases the tolerance of the ischemic kidneys against sustained I/R injury [2-4]. The IPC protective effect has been linked to various mechanisms such as decrease of inflammatory response [5], reduction of cellular apoptosis [6] and preservation of energetic pool [7]. Recent reports demonstrated that endoplasmic reticulum (ER) stress could play a critical role in acute kidney injury in humans and in animal model of I/R injury [8]. The stressed ER initiates the unfolded protein response (UPR) in order to reestablish the ER homeostasis [9,10]. However, when the ER stress is excessive, cell death is activated [1,11,12]. Recent studies demonstrated that IPC ameliorated I/R injury in brain [13] and heart [14] through ER stress diminution. However, its impact in ER stress in ischemic kidneys remains unknown.

Several mediators have been described to contribute to the protective effects of IPC, such as adenosine [15], bradykinin [3], and reactive oxygen species [7]. In addition, Chen et *al*. suggested that IPC increased the pro-survival Akt signaling pathway [2] and enhanced eNOS expression [16]. It has been reported that NO plays an important role in both initiating and mediating IPC [2]. In agreement with this, reported studies proved that exogenous addition of an NO donor can reproduce the protective effect of IPC [15]. By contrast, a non-selective NO blocker aggravated renal damage [2,17]. Additionally, the HIF-1α has emerged as a key regulator of the molecular hypoxic response [18,19]. Recent study reported that HIF-1α accumulation by pharmacological preconditioning protects the kidney against acute ischemic injury [20]. Semenza et *al*. showed in their study that delayed IPC attenuates both morphological and functional injuries and such protective effect may be related to the increased expression of HIF-1α [21].

In the current study, we firstly investigated the relationship between the early renal IPC and HIF-1α expression after renal I/R and the possible involvement of NO in this relation. Secondly, we showed the relevance of this IPC/HIF-1 α system in the modulation of ER stress.

## Materials and methods

### Surgical Procedure

The study was performed with male Wistar rats weighing between 200-250 g. It respected the European Union regulations (Directive 86/609/CEE) for animal experiments. All animals (including those of sham group) were anesthetized with an intraperitonial injection of ketamine (50 mg/kg) and were placed in a supine position on a heating pad to maintain the body temperature at 37°C. A midline laparatomy was performed, and the vein and the artery of the left and the right kidneys were isolated. In addition, catheters were inserted (i) into the jugular vein, for administration of mannitol (10%) and heparin (50 U/ml), (ii) into the carotid artery, for measurement of blood pressure (Press Monitor BP-1, WPI, USA) and for blood samples collection and (iii) into the bladder, for collection of urine samples. To induce renal ischemia, the renal pedicles were occluded with non traumatic vascular clamps. Reperfusion was initiated by removal of the clamps. At the end of experiments, both kidneys were removed under fully maintained anesthesia.

### Experimental groups

**Figure 1:**
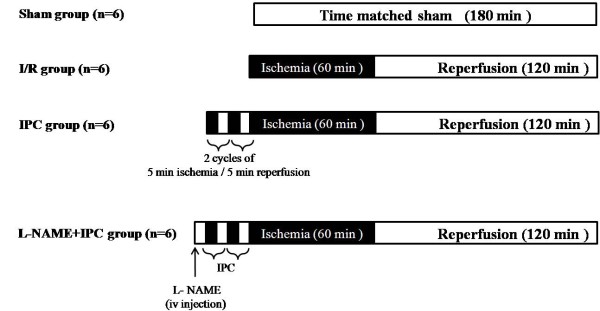
**Experimental protocol**. A schematic drawing of the experimental protocol used to determine the effect of ischemic preconditioning (IPC, 2 cycles of 5 min of ischemia (I) and 5 min of reperfusion (R)), and the effect of intravenously injection of N^G^-nitro-L-arginine methylester (L-NAME, 5 mg/kg) 5 min before IPC (L-NAME+IPC group). In sham group, rats underwent only dissection of renal pedicles; in I/R group, rats were subjected to 60 min of bilateral renal ischemia followed by 120 min of reperfusion (n = 6 in each group).

Animals were randomly divided into 4 experimental groups (n = 6 for each one, Figure 1): *I/R group*, renal pedicles were clamped for 60 min and then reperfused for 120 min. *IPC group*, just before the sustained ischemia, kidneys were subjected to 2 cycles of 5 min of ischemia followed by 5 min of reperfusion. Soon after, renal pedicles were clamped for 60 min then reperfused. *L-NAME+IPC group*, 5 min before IPC application, a non selective NOS inhibitor N^G^-nitro-L-arginine methylester (L-NAME, 5 mg/Kg) was intravenously administered to animals which were treated similar to IPC group. The results of all these groups were compared to those of a Sham group in which animals underwent only dissection of renal pedicles.

### Renal function

Blood and urine samples were collected to determine creatinine and sodium concentration. Creatinine concentrations in plasma and urine were measured according to the Jaffe's reaction (BioMerieux Kit, France). Sodium concentrations in plasma and urine were evaluated by a flame photometer (BT.634, Biotecnica instruments, Italy). Renal glomerular function was assessed by creatinine clearance.

The formula to calculate the creatinine clearance (μl/min/g) was: (Creat_u _. V)/Creat_p_

Creat_p_: creatinine concentration in plasma (μmol/l).

Creat_u_: Creatinine concentration in urine (μmol/l).

V: urine flow (μl/min/g).

Renal tubules function was evaluated by sodium reabsorption rate. The formula to calculate the sodium reabsorption rate (%) was: 100-[100 . (Na_u _. Creat_p_)/Na_p _. Creat_u_]

Na_u_: Sodium concentration in urine (mmol/l).

Na_p_: Sodium concentration in plasma (mmol/l).

Créat_p_: Creatinine concentration in plasma (μmol/l).

Créat_u_: Creatinine concentration in urine (μmol/l).

### Determination of lactate dehydrogenase activity

Lactate dehydrogenase (LDH) activity in plasma was used to evaluate kidney injury. The LDH activity was quantified using a standard kit (BioMerieux Kit, France).

### Determination of lipid peroxidation

Lipid peroxidation, used as an indirect index of the oxidative injury induced by the reactive oxygen species, was determined by measuring the formation of MDA with the thiobarbiturate reaction [22].

### Determination of nitrite and nitrate

Nitric oxide (NO) production in kidney was determined by tissue accumulation of nitrite and nitrate, as previously described [23].

### HIF 1α determination

Tissue HIF-1α concentration was quantified by binding of HIF-1α to its specific oligonucleotide containing the hypoxia response element, using the Trans AM HIF-1α kit (Active Motif, Carlsbad, CA, USA). Results are expressed as μg HIF-1α/mg protein [24].

### Western blot assay

The renal tissues were homogenized as previously described [25]. Proteins were separated by sodium dodecyl sulfate polyacrylamide gel electrophoresis and transferred into polyvinyldene fluoride membranes. Membranes were immunoblotted with antibodies directed against GRP78, ATF4, TRAF2, total and phosphorylated-PERK (Santa Cruz Biotechnology, Santa Cruz, CA, USA), total and phosphorylated Akt (Cell Signaling Technology Inc., Beverly, MA, USA), eNOS and β actin (Sigma Chemical, St. Louis, MO). The band of proteins was detected by using a chemiluminescent kit (Bio-Rad Laboratories, Hercules, CA, USA).and band intensities were quantified by densitometric scanning and the Quantity One software program (Bio-Rad Laboratories, Hercules, CA, USA).

### Statistical analysis

Data are expressed as mean ± SE (n = 6 for each group), and were compared statistically by variance analysis followed by the Student-Newman-Keuls test (Graph Pad Prism software).

*P *< 0.05 was considered significant.

## Results

### IPC reduced lipid peroxydation and cytolysis and improved renal function

**Figure 2:**
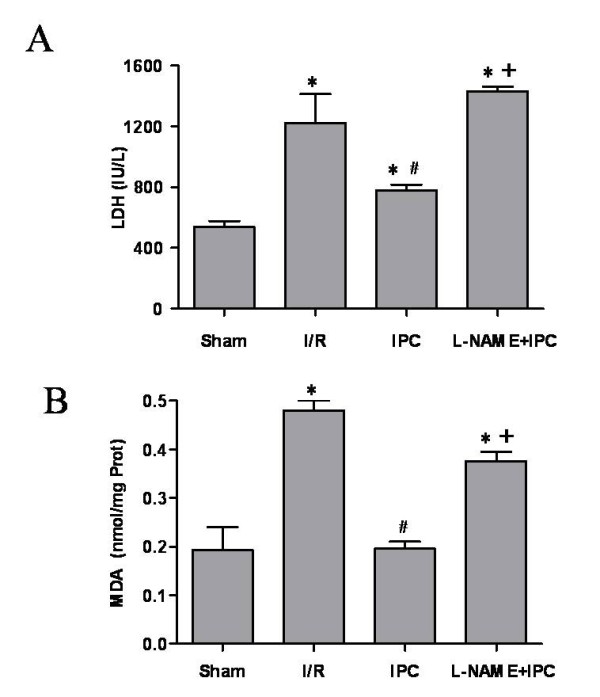
**Evaluation of lactate dehydrogenase activity in plasma (A) and malonedialdehyde concentration in tissue (B)**. Sham group: rats underwent only dissection; I/R group: renal pedicles underwent 60 min of warm ischemia (I) followed by 120 min of reperfusion (R). IPC group: renal pedicles underwent 2 cycles of 5 min of ischemia and 5 min of reperfusion, just before sustained ischemia. L-NAME+IPC group: 5 min before IPC, 5 mg/kg of N^G^-nitro-L-arginine methylester (L-NAME) was intravenously administered. Results are presented as mean ± SEM (n = 6 in each group). *P < 0.05 vs. Sham group, ^#^*P *< 0.05 vs. I/R group, ^+^*P *< 0.05 vs. IPC group.

**Figure 3:**
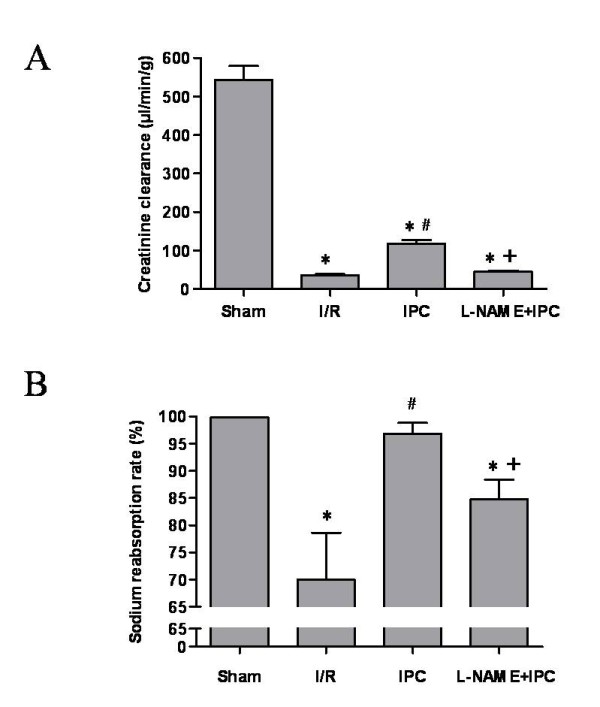
**Evaluation of creatinine clearance (A) and sodium reabsorption rate (B)**. Sham group: rats underwent only dissection; I/R group: renal pedicles underwent 60 min of warm ischemia (I) followed by 120 min of reperfusion (R). IPC group: renal pedicles underwent 2 cyles of 5 min of ischemia and 5 min of reperfusion, just before sustained ischemia. L-NAME+IPC group: 5 min before IPC, 5 mg/kg of N^G^-nitro-L-arginine methylester (L-NAME) was intravenously administered. Results are presented as mean ± SEM (n = 6 in each group). *P < 0.05 vs. Sham group, ^#^*P *< 0.05 vs. I/R group, ^+^*P *< 0.05 vs. IPC group.

As depicted in Figure 2, IPC application improved cell integrity and decreased lipid peroxydation as compared to I/R group. We found 780 ± 37 vs 1224 ± 191 IU/L (p < 0.05) and 0.20 ± 0.01 vs 0.48 ± 0.02 nmol/mg prot (p < 0.05) for plasma LDH activity and tissue MDA concentration, respectively. Subsequently, IPC statistically enhanced functional parameters of ischemic kidneys when compared to I/R group (Figure 3). Indeed, we noted 118 ± 9 vs 36 ± 3 μL/min/g for creatinine clearance and 96.8 ± 1.9 vs 70.0 ± 8.6% for sodium reabsorption rate. The L-NAME administration abolished the protective effects of IPC and no statistical differences were observed between I/R and L-NAME+IPC groups regarding all these parameters (p > 0.05).

### IPC increased pAkt, eNOS, nitrite/nitrate and HIF 1α levels

**Figure 4:**
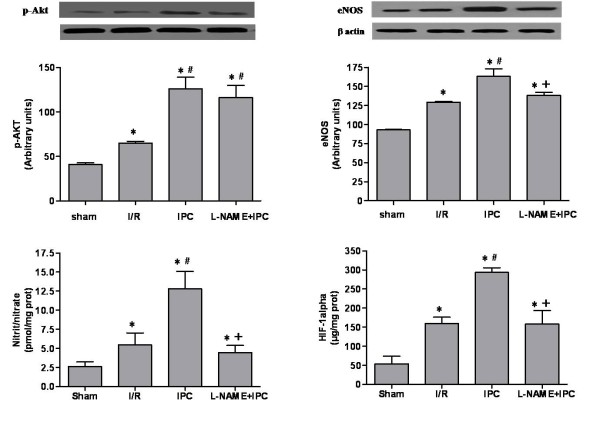
**p-Akt, endothelial nitric oxide synthase, nitrite/nitrate and HIF1-α**. A: Representative western blotting of p-Akt (upper panels) and densitometric analysis (lower panels); B: Representative western blotting of endothelial nitric oxide synthase (eNOS) (upper panels) and densitometric analysis (lower panels); C: Nitrite/nitrate levels; D: Hypoxia-induced factor-1α (HIF-1α) protein levels. Sham group: rats underwent only dissection; I/R group: renal pedicles underwent 60 min of warm ischemia (I) followed by 120 min of reperfusion (R). IPC group: renal pedicles underwent 2 cyles of 5 min of I and 5 min of R, just before sustained I. L-NAME+IPC group: 5 min before IPC, 5 mg/kg of N^G^-nitro-L-arginine methylester (L-NAME) was intravenously administered. Results are presented as mean ± SEM (n = 6 in each group). *P < 0.05 vs. Sham group, ^#^*P *< 0.05 vs. I/R group, ^+^*P *< 0.05 vs. IPC group.

The I/R injury resulted in a significant (p < 0.05) rise in p-Akt level compared to sham group (Figure 4). This level was further enhanced after IPC treatment (p < 0.05 vs I/R group). Additionally, IPC increased eNOS activation when compared to I/R group (p < 0.05). This in turn resulted in a significant (p < 0.05) increase in tissue nitrite/nitrate level. We found 13 ± 2 pmol/mg prot and 5.5 ± 1.5 pmol/mg prot respectively for IPC and I/R group. Moreover, our results showed that IPC application promoted the stabilization of HIF1α as referred to I/R. We observed 293 ± 13 μg/mg protein for IPC group and 160 ± 16 μg/mg prot for I/R group (p < 0.05).

The use of L-NAME before IPC did not affect the results of p-Akt compared to IPC group. However, it has declined significantly the level of eNOS (138 ± 4, p < 0.05 vs IPC group), the concentration of nitrite/nitrate (4.5 ± 0.9 pmol/mg prot, p < 0.05 vs IPC group) and the level of HIF-1α (158 ± 35 μg/mg prot, p < 0.01 vs IPC group) as referred to IPC group. It should be noted that no statistical difference (p > 0.05) between L-NAME+IPC and I/R groups was found for the amounts of eNOS, nitrite/nitrate and HIF-1α (Figure 4).

### IPC reduced ER stress

**Figure 5:**
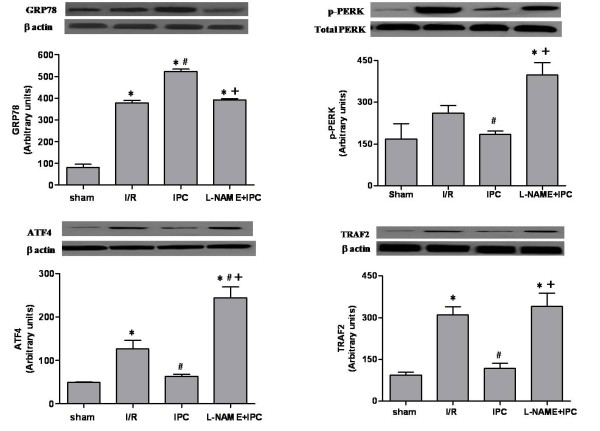
**Western blot of GRP78 (A), total and phosphorylated-PERK (B), ATF4 (C) and TRAF2 (D) protein levels**. The upper panels show one representative blot of six independent experiments and the lower panels show densitometric analysis. Sham group: rats underwent only dissection; I/R group: renal pedicles underwent 60 min of warm ischemia (I) followed by 120 min of reperfusion (R). IPC group: renal pedicles underwent 2 cyles of 5 min of I and 5 min of R, just before sustained I. L-NAME+IPC group: 5 min before IPC, 5 mg/kg of N^G^-nitro-L-arginine methylester (L-NAME) was intravenously administered. Results are presented as mean ± SEM (n = 6 in each group). *P < 0.05 vs. Sham group, ^#^*P *< 0.05 vs. I/R group, ^+^*P *< 0.05 vs. IPC group.

As indicated in Figure 5, our results showed an increase in GRP78 (p < 0.05), in p-PERK, in ATF4 (p < 0.05) and in TRAF2 (p < 0.05) after renal I/R compared to sham group respectively. Interestingly, IPC improved ER homeostasis. This was concomitant with a significant (p < 0.05) increase of GRP78 level and a reduction of the relative amounts of PERK, ATF4 and TRAF2 as judged to I/R group (p < 0.05 respectively). However, the treatment with L-NAME abolished the impact of IPC on ER stress parameters.

## Discussion

The detrimental effects of renal I/R injury are now well recognized. Interestingly, IPC has been shown to protect multiple organs [13,14,26] including kidney [15] from I/R injury in animals. In humans, some investigations have demonstrated the usefulness of IPC in cardiac [27], and liver surgery and transplantation [28,29], but no studies on human kidneys have been carried out [30]. The molecular bases of IPC involve the liberation of several mediators that afterwards take place on a multiple and complex intracellular signal pathways [31,32]. There is a biphasic temporal relationship between IPC-induced protection and the duration of reflow [33]. It offers an initial protection during 2-3 hours after reperfusion and a remote protection at the 12-24 hours that lasts for 2 to 3 days. In fact, this acute window was defined as the strongest form of *in vivo *protection against myocardial I/R [30,34]. This early window activates various signal transduction pathways and second messengers which then, serve as signals responsible for activation of the molecular and genetic responses of the delayed IPC [35]. In the current study, we were interested to explore the protective effect of IPC during early reperfusion.

Among the numerous signaling pathways involved in the protective mechanism of IPC, Akt signaling pathway plays a crucial role in defending various organs against I/R injury. Joo et *al *showed that acute renal IPC is associated with rapid phosphorylation of the Akt and that the inhibition of Akt pathway blocked the protective effects of renal IPC [33]. In agreement with this, our results revealed a marked increase of the phosphorylated form of Akt after IPC in comparison to I/R group. Strong evidences showed that eNOS is an important target of p-Akt [36,37]. Taken together, our results showed that IPC increased Akt phosphorylation, which in turn activated eNOS and NO generation to alleviate the I/R injury of ischemic kidneys.

It has emerged that NO plays a key role in triggering IPC phenomenon in different organs via its antioxidant [2], anti-apoptotic [34] and anti inflammatory properties [15]. It is known that inducible NOS (iNOS) activity was not detected before and immediately after ischemia, but it gradually increased after reperfusion [6]. In kidney, a previous investigation has showed that the expression and the activity of iNOS increase 24 h after IPC application [33]. Besides, it has been observed that pharmacological inhibition of NO synthesis or genetic deletion of the iNOS gene augments mouse kidney susceptibility and mitigates the protection afforded by IPC [15]. From these studies, the increase of iNOS expression could be considered as an important component of long-term protection induced by the IPC in the kidney. Nevertheless, eNOS-mediated NO production plays a crucial role in the acute preconditioned kidney [6]. Interestingly, our study showed that IPC resulted in the increase of nitrite/nitrate level which was markedly higher than this of I/R group. However, pharmacological inhibition of NOS with L-NAME abolished these beneficial effects of IPC against renal I/R injury. These results may reflect the critical role of NO and eNOS, at least partially, to trigger mechanisms responsible for inducing the protective effect of the early IPC.

Several regulatory molecules have been described to activate various signaling pathways able to reduce renal I/R injury. For instance, pharmacological up-regulation of HIF-1α represents a novel strategy in the prevention of acute kidney injury [19]. HIF-1α, a master regulator of gene transcription, mediates many processes of adaptation to low oxygen tension during hypoxia and ischemia [24]. Nevertheless under normoxic conditions, degradation of HIF-1α is mediated by oxygen-dependent hydroxylation of specific prolyl residues of the regulative alpha-subunits by HIF prolyl hydroxylases (PHD). It was demonstrated that inhibition of HIF-1α degradation by pharmacologic inhibition of PHD would confer protection against subsequent ischemic injury [18]. Recently, it was proved that renal hypoxic conditioning increased the expression of HIF-1α which correlated with the decrease of oxidative stress [1,38]. In line with this, our results clearly indicate that IPC application increased the level of HIF-1α as compared to I/R. We found that the IPC enhanced the stabilization of HIF-1α to protect ischemic kidneys against reperfusion injury. However, L-NAME treatment abolished this effect. Such results may reflect the involvement of NO in the preservation of HIF-1α stability in ischemic kidney. Zaouali et *al*. demonstrated that NO could favor the stabilization of HIF-1α in the liver [24]. Based on these findings and our results, it thus seems that IPC ameliorates kidney tolerance against I/R injury throughout enhancement of NO induced HIF-1α stabilization.

The involvement of ER stress in renal cells has been shown to be critical in acute kidney injury in humans and in various animal models of I/R injury [39,40]. Several factors including oxidative stress, disturbance of calcium homeostasis, and over expression of normal and/or incorrectly folded proteins, interfered with ER function and induced ER stress [41]. The stressed ER triggered the UPR which induces signal transduction events to increase ER resident chaperones, to inhibit protein translation, and to accelerate the degradation of unfolded proteins [10]. Our results showed a marked decline in the levels of p-PERK, ATF4, and TRAF2 and an increase of GRP78 level in IPC group. Studies performed during the last decade identified GRP78 as a ubiquitous luminal resident protein of the ER that plays a key role in assisting the corrected folding and secretion of protein [42]. This protein can protect cells from ER stress and its induction is crucial for maintaining the viability of cells subjected to stress [43,44]. Hayashi et *al*. found that induction of GRP78 by IPC reduced ER stress and prevented delayed neuronal cell death [41]. Moreover, Hung et *al*. observed that blocking the induction of GRP78 sensitized the renal epithelial cells to oxidative stress. Therefore, we could suggest that IPC protected kidneys against renal I/R insults throughout modulation of ER stress and that up regulation of GRP78 made kidneys more resistant to the stressful conditions. Nevertheless, this beneficial effect was abolished when L-NAME had been administered before IPC treatment. In line with this, Bachar et *al*. proved in pancreatic beta cells subjected to glucolipotoxic conditions, that chronic inhibition of NO production exacerbates ER stress [45]. Taken together, our results revealed that IPC modulated ER stress through the activation of the eNOS pathway, since NOS inhibitor counteracted the protective effect of IPC to reduce ER stress. In addition, Xu et *al*. strongly suggest in their study that the NO increased the GRP78 expression. They proposed that NO regulates the flux of Ca^2+ ^between the mitochondria, the Golgi and the ER. This results in activation of the ER-stress response transcription factor ATF6 which then translocates to the nucleus and activates ER stress-responsive genes, such as GRP78 [46].

## Conclusions

In conclusion, we found that IPC early protects kidney against renal I/R injury via reducing oxidative and ER stresses. These effects are associated with phosphorylation of Akt and activation of eNOS and also with stabilization of HIF-1α. The beneficial impact of IPC is abolished when NO production is inhibited before IPC application.

## List of abbreviations

ATF4: activating transcription factor 4; ER: endoplasmic reticulum; eNOS: endothelial nitric oxide synthase; GRP78: glucose-regulated protein 78; HIF-1α: hypoxia-inducible transcription factor-1α; iNOS: inducible nitric oxide synthase; I/R: ischemia reperfusion; IPC: ischemic preconditioning; LDH: lactate dehydrogenase; L-NAME: N^G^-nitro-L-arginine methylester hydrochloride; MDA: malonedialdehyde; NO: nitric oxide; PERK: RNA activated protein kinase (PKR)-like ER kinase; PHD: prolyl hydroxylases; TRAF2: TNF-receptor-associated factor 2; UPR: unfolded protein response.

## Competing interests

The authors declare that they have no competing interests.

## Authors' contributions

AMB and MAZ carried out the experimental work and analyzed data. AMB, HBA and JRC designed the study, coordinated the experiments, analyzed data and wrote the manuscript. KHA conducted the statistical analyses. AHM conducted the biochemical analyses. DSM, analyzed the data. All authors read and approved the final manuscript.
